# The expected burden of mesothelioma mortality in Great Britain from 2002 to 2050

**DOI:** 10.1038/sj.bjc.6602307

**Published:** 2005-01-25

**Authors:** J T Hodgson, D M McElvenny, A J Darnton, M J Price, J Peto

**Affiliations:** 1 Epidemiology and Medical Statistics Unit, Health and Safety Executive, Magdalen House, Trinity Road, Bootle, Merseyside L20 3QZ, UK; 2 Department of Epidemiology and Population Health, London School of Hygiene and Tropical Medicine, Keppel Street, London WC1E 7HT, UK; 3 Epidemiology Section, Institute of Cancer Research, 15 Cotswold Road, Belmont, Sutton, Surrey SM2 5NG, UK

**Keywords:** mesothelioma, mortality, Great Britain, prediction

## Abstract

The British mesothelioma register contains all deaths from 1968 to 2001 where mesothelioma was mentioned on the death certificate. These data were used to predict the future burden of mesothelioma mortality in Great Britain. Poisson regression analysis was used to model male mesothelioma deaths from 1968 to 2001 as a function of the rise and fall of asbestos exposure during the 20th century, and hence to predict numbers of male deaths in the years 2002–2050. The annual number of mesothelioma deaths in Great Britain has risen increasingly rapidly from 153 deaths in 1968 to 1848 in 2001 and, using our preferred model, is predicted to peak at around 1950 to 2450 deaths per year between 2011 and 2015. Following this peak, the number of deaths is expected to decline rapidly. The eventual death rate will depend on the background level and any residual asbestos exposure. Between 1968 and 2050, there will have been approximately 90 000 deaths from mesothelioma in Great Britain, 65 000 of which will occur after 2001.

Mesothelioma is a formerly rare cancer that principally affects the pleura and the peritoneum (Greenberg and Lloyd Davies, 1974) and is almost always caused by asbestos exposure. The disease is rapidly fatal, most of those affected dying within a year of diagnosis (Peto *et al*, 1995). There is a long latent period between first exposure to asbestos and diagnosis of mesothelioma that is seldom less than 15 years and often exceeds 60 years (Bianchi *et al*, 1997). In all, 85% of deaths are among men, and the risk is highest in occupations with substantial exposure to asbestos (McElvenny *et al*, 2005).

Previous predictions of future numbers of mesothelioma deaths in Britain were based on observed male deaths between 1968 and 1991 (Peto *et al*, 1995; Hodgson *et al*, 1997) and were derived from a simple birth cohort model in which mesothelioma risk was related independently to age and to date of birth. These analyses suggested that male mesothelioma deaths would peak at about 2700–3300 deaths around 2020. The data have conformed to this model well up to 1991, but from the 1990s show a departure from fit for some of the later cohorts (Figure 1A and B). The slower increase with age for mesothelioma death rates in the 1990s implies that the simple age–cohort model is not reliable. We fitted the age–cohort model as confirmation of these observations, but our main models related current mortality to past asbestos exposure levels and did not assume the same age distribution of mortality in different birth cohorts.

## MATERIALS AND METHODS

The mesothelioma register is described in detail elsewhere (McElvenny *et al*, 2005). Briefly, deaths occurring in England, Scotland and Wales, where mesothelioma was mentioned on the death certificate, are notified annually to the Health and Safety Executive by the Office for National Statistics and the General Register Office for Scotland. Originally, this was done manually as part of the coding of causes of death, but since 1992 (1996 in Scotland), the computerised national mortality database has been scanned. Checks for completeness include searching deaths in England and Wales for alternative spellings of mesothelioma and flagging cancer registrations with a morphology code consistent with mesothelioma for death notification at the National Health Service Central Registers in Edinburgh and Southport.

Details of the analysis, which was based on deaths in men aged 20–89 years between 1968 and 2001, are given in Appendix A1. The model parameters were estimated iteratively, and the deviance (based on observed and expected numbers aggregated over 5-year groups by age, date of birth and year of death) was used to assess overall goodness of fit. Two of the model parameters, *k* (the power of time defining the increase in risk after exposure) and *H* (the half-life for clearance of asbestos from the lung), are closely correlated and cannot be independently estimated. The effect of reducing the half-life is to increase the best-fit value of *k*, but the fit of the model is affected only slightly. Since fitting the model with both *H* and *k* is unstable, we used two versions of the model, one with (effectively) no clearance (*H*=1000 years) and the other with a clearance half-life of 15 years – a value suggested from the modelling of mortality of the Wittenoom workforce (Berry, 1991).

The adequacy of the model was tested by examining deviance residuals and by comparing observed and fitted numbers of mesothelioma deaths. The uncertainty of the estimate was quantified by calculating an approximate 95% confidence (CI) interval for the peak in predicted future number of mesothelioma deaths and the year in which this peak was expected to occur. Our estimate of the predicted peak was scaled to include mesothelioma deaths in men aged 90 years or older, and deaths among women.

Mesothelioma mortality is very low up to 20 years after first asbestos exposure even among heavily exposed workers (Peto *et al*, 1982), so death rates up to 2001 give virtually no information on exposures in the previous 20 years. The level and timing of the predicted peak in mortality are virtually unaffected by exposure since 1980, but for longer-term prediction recent exposures become increasingly relevant, and some assumption must be made about asbestos exposure since the 1970s. An assessment of current exposure levels for a recent HSE Regulatory Impact Assessment (HSE, 2002) suggested that population exposure in the 2000 was around 4% of the peak value reached in the 1960s. For the present projections, we assumed a continuing decline in asbestos exposure, from 4% of the peak level in 2000 to 2% by 2010 and 0.75% by 2050.

## RESULTS

**Figure 1:**
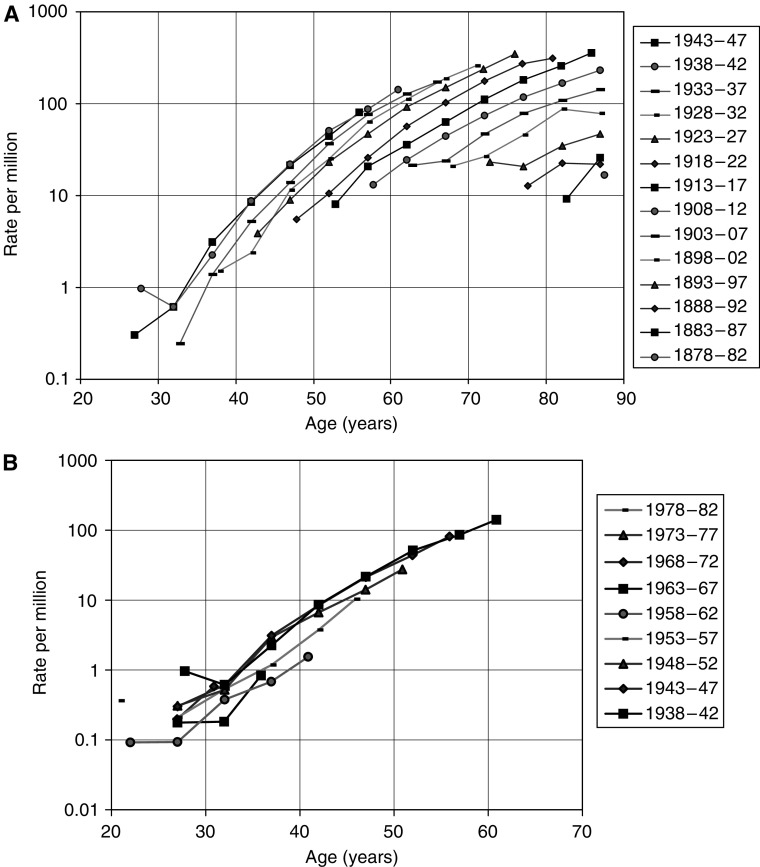
(**A**) Mesothelioma death rates by 5-year cohort and age group for males born 1878–1937 (note: The earlier cohorts are to the lower right of the chart). (**B**) Mesothelioma death rates by 5-year cohort and age group for males born 1938–1982 (note: The earlier cohorts are to the upper left of the chart).

**Figure 2:**
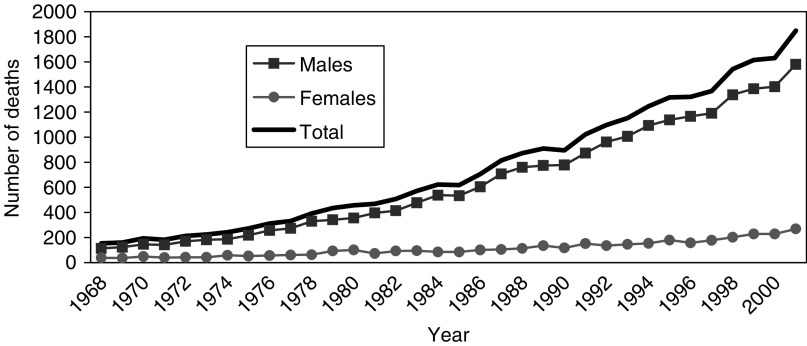
Mesothelioma deaths by sex and year.

**Figure 3:**
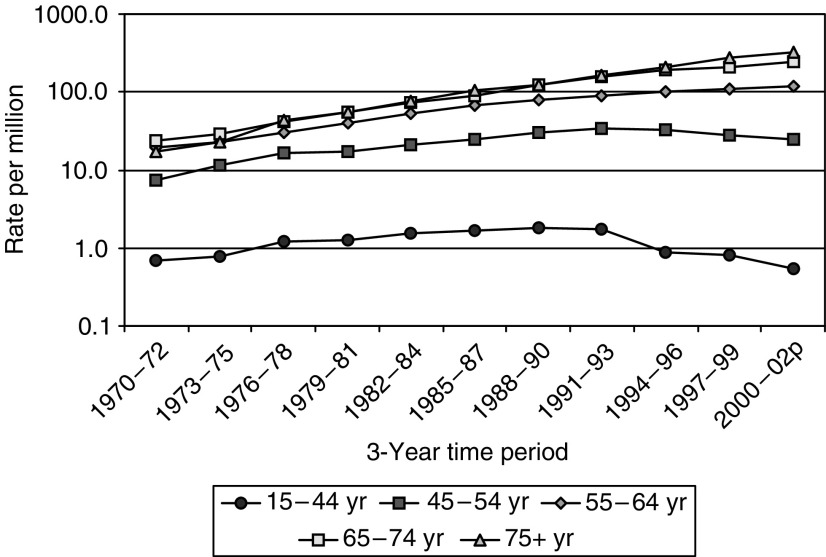
Average annual male mesothelioma death rates per million by age and time period.

The annual number of mesothelioma deaths in Great Britain has risen increasingly rapidly by about 12-fold from 1968 (the first year in which ascertainment is believed to be complete) to 2001 (Figure 2). The mesothelioma death rate in males has continued to increase in older age groups, but has decreased among younger men in recent years (Figure 3). In 2001, there were 1579 male deaths, 85% of the total, the majority being at ages 60–79 years, with relatively few aged less than 50 years. Less than 1% of deaths in men have occurred at age 90 or older, but the number is likely to increase substantially over time. Men born around 1940 have suffered higher death rates than any previous or subsequent birth cohort (Figures 1A and B), and they will not reach 90 years of age until 2030.

**Figure 4:**
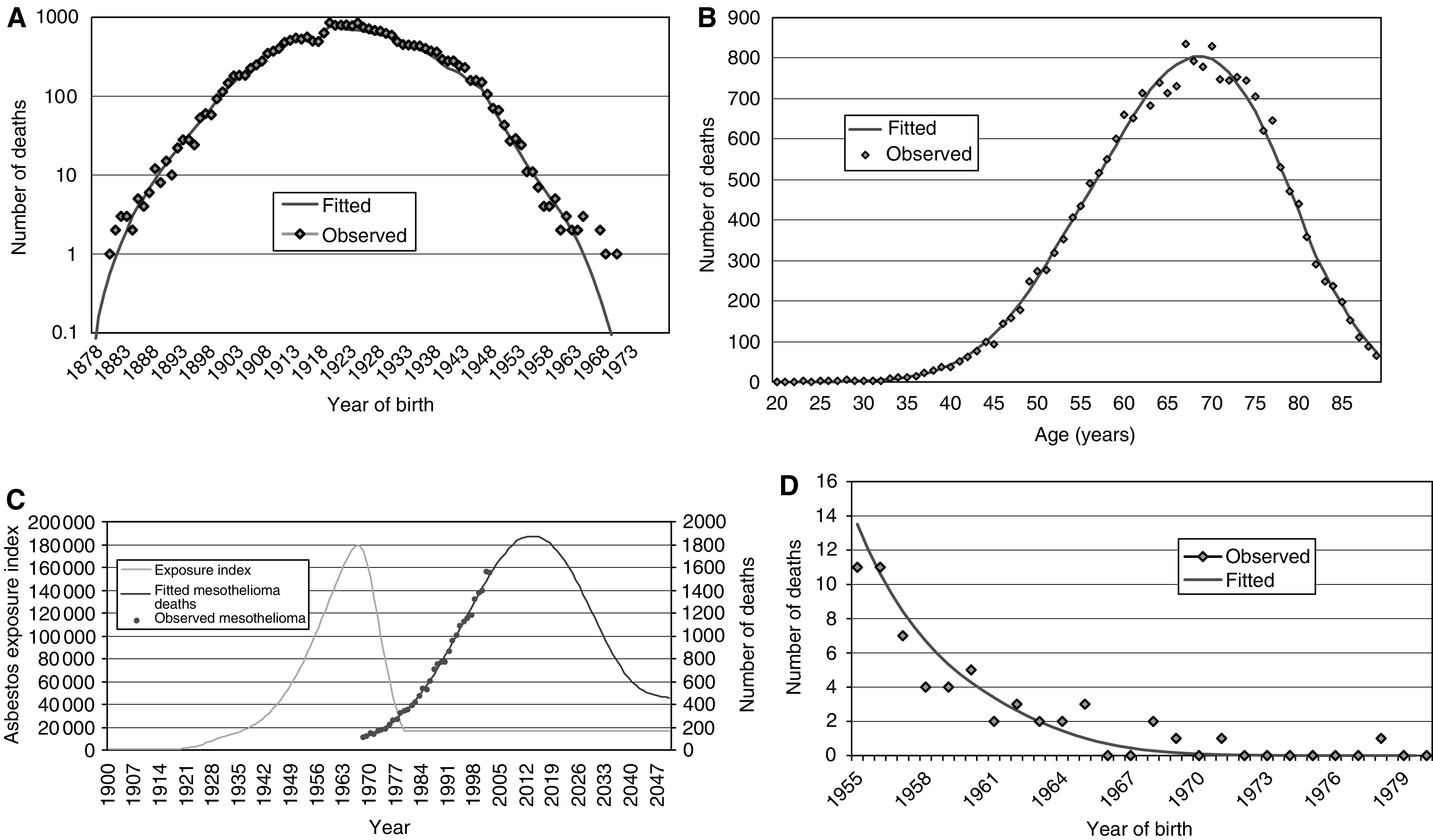
(**A**) Observed and fitted deaths by year of birth. (**B**) Observed and fitted deaths by age. (**C**) Observed and fitted mesothelioma deaths by year of death, with derived exposure index. (**D**) Number of observed and fitted mesothelioma deaths for 1955–1980 yearly birth cohorts.

Parameter values estimated from the two versions of the Poisson regression model are shown in the Table 1. The two versions of the model produced effectively equivalent fits overall, and showed very similar patterns of residuals. This is illustrated (for the nonclearance model) in Figures 4A–C which show plots of fitted and observed deaths by year of birth, age and year of death. These show close agreement. A two-dimensional array (not shown) of deviance residuals by 5-year age and year of birth groupings also showed an unbiased pattern with no strong clustering of residuals of the same sign. While the fit of the clearance and nonclearance versions of the model to past data were equivalent, their predictions of future mortality were different. Under the nonclearance model, the annual number of mesothelioma deaths in men aged below 90 years is predicted to reach a peak of 1846 deaths (95% CI 1650–2100) in 2013 (95% CI 2011–2015). The clearance model predicts a peak of 1983 deaths (95% CI 1835–2233) in 2011 (95% CI 2009–2013). As more data accumulate on the pattern of reduced mortality related to exposure levels after the mid-1960s peak, it may be possible to estimate the parameters *k* and *H* separately.

Allowing for deaths occurring in men aged 90 or over, the estimated peak among males is 1857. Assuming a linear relationship between annual numbers of male *M* and female *F* mesothelioma deaths (*F*=22.9+0.136*M*; *R*^2^=0.95), the maximum number of female deaths was estimated to be 276, giving a predicted total of 2133 mesothelioma deaths (95% CI 1950–2450) at the peak of the epidemic. The total number of mesothelioma deaths to 2050 is predicted to be around 90 000, with 65 000 of these occurring from 2002 onwards.

## DISCUSSION

The age–cohort model was fitted (residual deviance 121.5 on 72 degrees of freedom) and confirmed the observed departure of the data from that model by providing a significantly worse fit than our preferred model (residual deviance 235.6 on 182 degrees of freedom). The existence of two versions of the model (one with clearance and the other with no clearance) with equivalent overall fits but with different future implications raises the obvious question as to which is closest to the truth.

**Figure 5:**
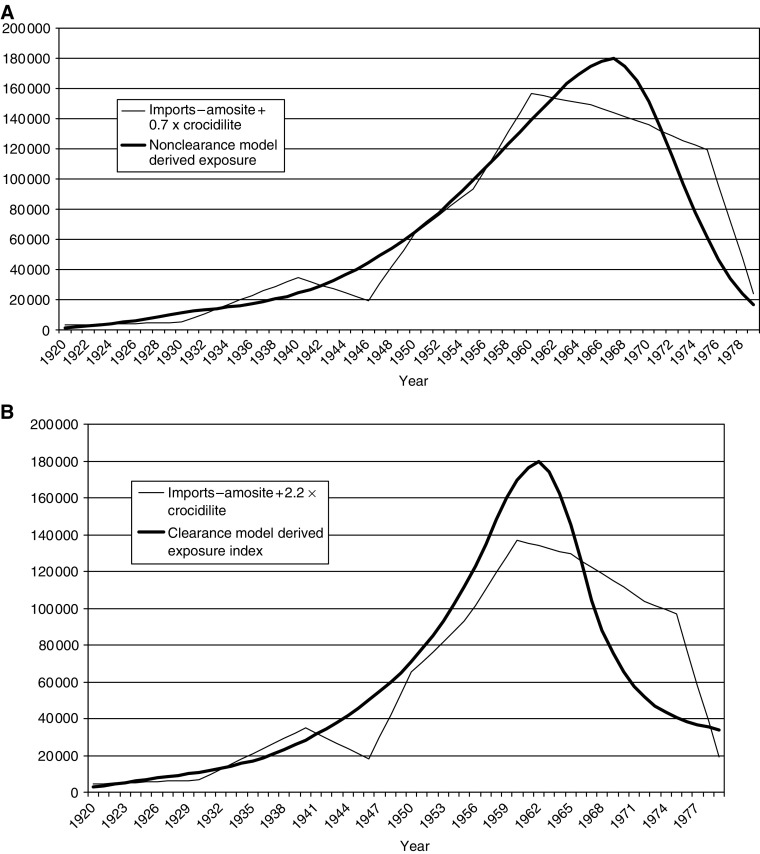
(**A**) Fitted import index for nonclearance model with best approximating weighting of actual import series (amosite+0.7 × crocidolite. (**B**). Fitted import index for clearance model with best approximating weighting of actual import series (amosite+2.2 × crocidolite).

One way of assessing which model is the most realistic is to match the implied exposure patterns from each of the models with the actual pattern of asbestos imports, taking account of the difference between fibre types. Figure 5A shows the fitted exposure index for the nonclearance model together with the best approximating linear combination of the three fibre-specific import profiles, and Figure 5B shows this information for the lung clearance model (chrysotile had zero weight in both). It is evident that overall approximation is less good for the clearance than for the nonclearance exposure index. Although this argument is not definitive, we interpret the better agreement of imports with the exposure profile associated with the nonclearance model as justifying a modest preference for this model in developing our current projections. This preference is reinforced by the fact that the observed 2001 total number of mesothelioma deaths is a more extreme outlier in relation to the clearance model (1563 observed *vs* 1463 expected, *P*=0.0022) than in relation to the nonclearance model (1488 expected, *P*=0.0143).

Our assumption that exposure in 2000 was 4% of the peak level hardly affects the predicted peak in mesothelioma mortality. It does, however, influence the predicted number of cases following the peak. Thus, the total burden of mesothelioma mortality to 2050 remains very uncertain. The estimated power of time since first exposure of 2.6 is in the range expected on the basis of fits of similar models to cohort data, providing a measure of support for the application of the model at that population level. The diagnostic trend, a decrease of 5% per year in the proportion of cases that are undiagnosed, implies that in 1968 about 90% of cases were diagnosed. This parameter improves the fit but has virtually no influence on the projections.

The model was fitted by minimising the total deviance. Recent birth cohorts have fewer mesothelioma deaths, so the fitted model is dominated by earlier cohorts with longer follow-up. The 1920–1924 cohort has the most deaths. Residuals for the most recent cohorts therefore provide an indicator of predictive reliability. Figures 4A and D show observed and fitted deaths by year of birth, with some divergence for the most recent births (Figure 4D). From 1965 onwards there are eight deaths compared to a fitted value of 3.5, a borderline significant excess (*P*=0.054). However, the fitted model does not include an allowance for a background rate of mesothelioma. If there were, as widely assumed, around 1–2% of male mesothelioma deaths not due to asbestos exposure, this equates to around 50 male deaths annually in Great Britain. On this basis, around four deaths would be expected among those not exposed to asbestos and born from 1965 onwards, bringing observed and fitted into good agreement. As data accumulates in these more recently born groups, it will be important to make explicit allowance in the model for the possibility of a background rate. In terms of projecting the timing and level of peak mortality, however, the numbers generated by this background rate can be ignored.

Predictions of the future number of mesothelioma cases have been attempted in other countries. For example, in Denmark, the 1912 cases of malignant mesothelioma reported to the Danish cancer registry between 1943 and 1993 were used to predict a peak incidence of 93 cases among men born before 1955 to around the year 2015 (Kjaergaard, 2000). In the United States, using mesothelioma incidence data from the Surveillance, Epidemiology and End Results programme, it has been estimated that there will be a peak around the years 2000 to 2004 of approximately 2000 cases and a return to background incidence by 2055 (Price and Ware, 2004). In Australia, the incidence of mesothelioma is expected to peak around 700 cases per year in 2010 (Leigh and Driscoll, 2003). In Sweden, the preventive measures of the mid-1970s can probably not be evaluated with reasonable precision until around 2005 (Jarvholm *et al*, 1999). In the Netherlands, it has been predicted that pleural mesothelioma will peak around the year 2028, with up to 900 cases per year (Segura *et al*, 2003). In France, the number of deaths is predicted to reach a peak at about 2200 per year some time after 2020 (Ilg *et al*, 1998). Based on combined data from Britain, France, Germany, Italy, the Netherlands, Switzerland and Hungary, it has been suggested that the number of men dying from mesothelioma will almost double over the next 20 years, peaking at about 90 000 cases around 2018 (Peto *et al*, 1999). These projections – like the earlier British projections (Peto *et al*, 1995) – were based on a simple age and birth cohort model. More recent death rates in three of these countries (France, Germany and Italy) are lower than were predicted, and the timing and level of the peak of Europe-wide mesothelioma deaths may prove to be earlier and lower, respectively, than previous projections suggested.

## CONCLUSIONS

The data for 2001 showed around 1850 mesothelioma deaths among males and females in Great Britain. Using a statistical modelling approach, mesothelioma mortality in Great Britain is predicted to peak at around 1950–2450 deaths per year some time between 2011 and 2015. Around 90 000 deaths are predicted to occur by 2050, with 65 000 of these occurring from 2002 onwards.

## Figures and Tables

**Table 1:** Estimated parameter values from optimal Poisson's regression model

**Parameter**	**Estimates for non clearance model (half life=1000 years)**	**Estimates for model with clearance half life of 15 years**
Power of time since first exposure, *k*	2.6	4.1
Year of exposure maximum	1967	1962
Diagnostic trend (percentage decrease in missed cases per year), *Dx*_*T*_	5	2

*Relative exposure potential for age group (years)*		
0–4	0.00	0.00
5–15	0.03	0.07
16–19	0.21	1.18
20–29	1.00 (baseline)	1.00 (baseline)
30–39	1.24	1.95
40–49	1.11	1.02
50–59	0.00	0.08
60–64	0.00	0.00
65+	0.00	0.00

*Change in exposure index (percentage per year) in peak year±X years*	% *change*	% *change*
−45	29	30
−35	6	8
−25	11	11
−15	9	9
−5	5	10
0 (peak year)	0	0
+5	−14	−17
+15	−39	−4

## Appendix A1

### THE POISSON REGRESSION MODEL

*Introduction* Mesothelioma mortality rises rapidly with increasing time since first asbestos exposure but is independent of age (Peto *et al*, 1982). These observations can be explained by a dose–response model in which the increase in subsequent mesothelioma risk caused by each episode of asbestos exposure is proportional to the cumulative dose inhaled and to the second or third power of time since the exposure (Peto, 1979). The fit is slightly improved up to 20 years after first exposure by assuming a lag of 10 years before mortality begins to increase (Peto *et al*, 1982). This relationship has been used as a basis for risk assessment and regulation by several agencies, including the United Kingdom (Doll and Peto, 1985) and the United States of America (Health Effects Institute, 1991). We have adapted the model to find an asbestos exposure distribution by year and by age that generates predicted mesothelioma deaths by year and age close to the observed pattern from 1968 to 2001, and hence to predict future rates. The model was developed to predict an individual's risk from his level of asbestos exposure in each previous year, but we have used it to predict the death rate in each birth cohort of British men as a function of their average exposure in each year.

*Model formulation* Under the dose–response model for mesothelioma developed by the Health Effects Institute (HEI) (Health Effects Institute, 1991), an individual's additional mesothelioma risk caused by each brief exposure to asbestos is proportional to the increase in cumulative exposure *D* multiplied by the second or third power of time *t* since the exposure lagged by 10 years:

As the predicted risk after a given interval is directly proportional to the exposure *D*, the HEI model can also be applied at the population level to death rates in each birth cohort, replacing an individual's asbestos exposure *D* in a given period by the corresponding average collective dose.

The following additional assumptions were incorporated in the model we have fitted:

Average asbestos exposure to the male population of Great Britain in each year is summarised by a single estimate, but exposure also varies with age. This age dependence for exposure is assumed to be the same in all past and future periods.
A trend in the completeness of mesothelioma diagnosis over time is included.
A half-life of 1000 years was arbitrarily assumed for the proportion of fibres remaining in the lung. (The shorter half-life of 15 years increased the estimated power *k* but gave similar goodness of fit and predictions.)

The mesothelioma death rate for men aged *A* in year *T* was approximated by the sum of the risks due to exposure in all previous years of their lifetime, excluding the most recent 10 years. For each of these individual years, the contribution to the predicted death rate was calculated as the product of the appropriate age-specific exposure factor, the overall population exposure index for that year and the lagged time interval to year *T* raised to the power *k*. The predicted number of mesothelioma deaths at age *A* in year *T* is then proportional to the sum of these risk contributions multiplied by the estimated proportion of mesothelioma cases in year *T* that were diagnosed, and the total population aged *A* in year *T* (i.e. the person-years for age *A* and year *T*). Rescaling so that the total fitted number of mesothelioma deaths in the period 1968–2001 is equal to the total observed number in the same period gives the predicted number of deaths for persons aged *A* in year *T*. The model can be represented mathematically as follows:

where *F*_*A,T*_ is the number of deaths at age *A* in year *T*; *W*_*A*_ is the age-specific exposure potential at age *A*; *D*_*T*_ is the overall population exposure in year *T*; *Dx*_*T*_ is the proportion of mesothelioma deaths in year *T* that are recorded; *L* is the lag period in years between exposure and disease occurrence; *H* is the half-life in years for asbestos clearance from the lungs; *k* is the exponent of time representing the increase of risk with increase of time since exposure; *P*_*A,T*_ is the person-years at risk for age *A* in year *T*; *M* is the total observed mesothelioma deaths 1968–2001; the content of {} is set to zero when negative; the summations indexed by *l* represent the cumulative effect at age *A* of the exposures at earlier ages; and *l* indexes years lagged from the risk year.

*Details of model parameters* Age-specific exposure potential *W*_*A*_ was defined by assigning nine parameters to the age groups 0–4, 5–15, 16–19, 20–29, 30–39, 40–49, 50–59, 60–64 and 65+ years. Age group 20–29 years was chosen as the baseline category. General exposure level in a given year *D*_*T*_ is the average ‘effective carcinogenic dose’ in the breathing zone of men aged 20–29 years . If crocidolite were (for example) five times more dangerous than amosite, its contribution to *D*_*T*_ per unit dose would be five times greater.

The exposure distribution was defined by growth and decline rates for years in multiples of 10 before and after the maximum exposure year (in which exposure growth/decline is zero). Growth rates for years intermediate between the 10-yearly values were determined by linear interpolation. Owing to the long latency of mesothelioma, exposures after 1980 could not be estimated. Based on an assessment of the current distribution of exposures (set out in detail in a recent HSE Regulatory Impact Assessment – HSE, 2002), it was assumed that the total asbestos exposure to the population in 2000 would be approximately 4% of the peak value. Using predicted building demolition rates from 2001 to 2050, it was estimated that exposure would be at 2% of the maximum by 2010 and 0.75% of the maximum by 2050.

Diagnosis was assumed to be essentially complete (98%) in 1997. The diagnostic trend *Dx*_*T*_ was parameterised as the annual percentage increase in the number of missed cases working backwards in time from this year.

Clearance of fibres from the lung was assumed to follow an exponential decline parameterised by its half-life *H*.

*Testing of model adequacy and uncertainty* The adequacy of the final model was assessed by examining deviance residuals and comparing plots of observed and expected mesothelioma deaths. Approximate 95% CIs for the level and the timing of the predicted peak in mesothelioma deaths were calculated by adjusting model parameters to produce a lower/earlier peak and a higher/later peak, corresponding to a change in the deviance from the optimal model equivalent to the 5% critical value of the *χ*^2^ distribution on the number of degrees of freedom in the model. The resultant ranges are roughly equivalent to 95% CIs, but are slightly narrower, since they cannot take account of uncertainties in the mathematical formulation of the model.
